# Developing Chloroplast Genomic Resources from 25 *Avena* Species for the Characterization of Oat Wild Relative Germplasm

**DOI:** 10.3390/plants8110438

**Published:** 2019-10-23

**Authors:** Yong-Bi Fu, Pingchuan Li, Bill Biligetu

**Affiliations:** 1 Plant Gene Resources of Canada, Saskatoon Research and Development Centre, Agriculture and Agri-Food Canada, 107 Science Place, Saskatoon, SK S7N 0X2, Canada; 2 Department of Plant Sciences, University of Saskatchewan, 51 Campus Drive, Saskatoon, SK S7N 5A8, Canada; lipingchuan@gmail.com (P.L.); bill.biligetu@usask.ca (B.B.)

**Keywords:** Crop wild relative, *Avena*, chloroplast genome, chloroplast gene, positive selection

## Abstract

Chloroplast (cp) genomics will play an important role in the characterization of crop wild relative germplasm conserved in worldwide gene banks, thanks to the advances in genome sequencing. We applied a multiplexed shotgun sequencing procedure to sequence the cp genomes of 25 *Avena* species with variable ploidy levels. Bioinformatics analysis of the acquired sequences generated 25 *de novo* genome assemblies ranging from 135,557 to 136,006 bp. The gene annotations revealed 130 genes and their duplications, along with four to six pseudogenes, for each genome. Little differences in genome structure and gene arrangement were observed across the 25 species. Polymorphism analyses identified 1313 polymorphic sites and revealed an average of 277 microsatellites per genome. Greater nucleotide diversity was observed in the short single-copy region. Genome-wide scanning of selection signals suggested that six cp genes were under positive selection on some amino acids. These research outputs allow for a better understanding of oat cp genomes and evolution, and they form an essential set of cp genomic resources for the studies of oat evolutionary biology and for oat wild relative germplasm characterization.

## 1. Introduction

Chloroplast (cp) genomics will play an important role in the characterization of crop wild relative (CWR) germplasm conserved in worldwide gene banks. Currently, many CWR collections are expanding to mitigate the threats of losing CWRs from climate change and other habitat disturbances and to conserve germplasm for plant breeding (e.g., see [1,2]). Thus, the need for CWR germplasm characterization is increasing. Most CWR germplasm has complex, polyploid, and/or uncharacterized genomes [3,4], and current tools based on nuclear genome sequences may not always be effective in identifying CWR germplasm and investigating its genetic variability [5,6]. Conserved CWRs are often misclassified or require taxonomic evaluation [7]. More informative barcoding [8] will be needed to distinguish among CWR accessions for specific traits or features. Acquired CWRs will be used to enhance the studies of plant biology and evolution (e.g., see [6,9]). The search for cp genes in conserved CWR will be increased for applications in genetic engineering [10]. Therefore, it is important to develop cp genomic resources for characterizing CWR germplasm.

Oat (*Avena* L.) is one of the most widely cultivated cereals and a valuable resource in many countries, both for human consumption and animal feed [11]. The genus *Avena* has up to 30 recognized species, including diploids, tetraploids, and hexaploids [7,12]. The cultivated hexaploid oat has 42 chromosomes, representing three different sets of nuclear genomes (A, C, and D) [6]. Currently, there are more than 31,000 accessions of oat wild relative germplasm conserved in more than 20 gene banks worldwide [13]. These genetic resources are known to harbor an important source of genetic variability [14] for oat genetic improvement through interspecific crossing and introgression [7,15,16]. Conserving and managing these wild relative accessions are challenging tasks [17]. However, there is no report so far on the research to develop oat cp genomic resources for characterizing these oat accessions [18], although several cp-based evolutionary studies of oat species were conducted [9,19,20,21,22,23]. This research has helped to provide insights into the maternal origins of oat genomes, confirming the general consensus in oat phylogeny [9,18,24].

The recent advances in next-generation sequencing have made the sequencing of organelle genomes more feasible than before [25,26,27,28]. We applied a multiplexed shotgun sequencing procedure to sequence the cp genomes of 25 *Avena* species with variable ploidy levels. In our companion paper [18], we extracted single nucleotide polymorphism (SNP), using the wheat cp genome as the reference and inferred maternal patterns of oat evolution through phylogenetic analyses. In this paper, we will perform various types of bioinformatics analysis to assemble all 25 cp genomes, annotate the genome assemblies, conduct comparative genomic analysis, and analyze sequence divergence and selective pressure for cp genes. It was our hope that these analyses would provide baseline information that could give us a better understanding of the oat cp genome and evolution and for oat wild relative germplasm characterization.

## 2. Results

### 2.1. Sequencing and Assembly

Four MiSeq runs generated a total of 95 million sequence reads for 25 *Avena* samples, each having 3.8 million sequence reads (Table 1). After removing sequence reads of poor quality (Q < 15 and read length <150 bp), an average of 83% high-quality sequence reads were obtained for these samples. Thus, each sample still had a sequence length ranging from 1200 to 2500 Mbp, with an average of 1817 Mbp, corresponding to an approximately 8900× to 18,890× cp genome coverage. Such high genome coverages made the cp genome assembly simpler, with the smallest numbers of contigs and scaffolds under proper k-mer coverage and size setting.

**Figure 1:**
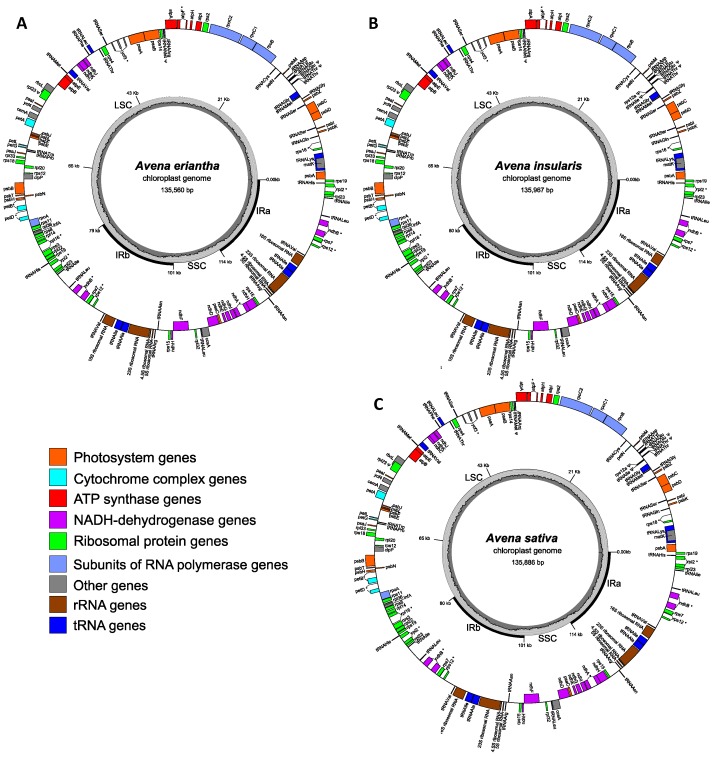
Gene maps of three selected *Avena* chloroplast genomes: (**A**) *A. eriantha* (2x), (**B**) *A. insularis* (4x), and (**C**) *A. sativa* (6x). Each map is represented in, moving counterclockwise from the right. The larger circle represents the layout of chloroplast genes distribution as per their transcription direction: outside boxes and inside boxes show the counterclockwise and clockwise transcription. The color of the gene box indicates the functional group that the gene belongs to. The smaller circle represents the CG content plot in the corresponding sample. LSC, large single copy region; SSC, small single copy region. IRa/b, inverted repeats. Intron-containing genes are marked by a ‘∗’ symbol and pseudogenes are marked by a ‘Ψ’ symbol.

*De novo* assembly with the pair-end sequence reads from each sample generated three major scaffolds, without any exception for all the 25 samples, as expected with two inverted repeats (IRs). As illustrated with 2x, 4x, and 6x oat species in Figure 1, all the circular *Avena* cp genomes consisted of four typical DNA fragment structures: one large single copy (LSC), one small single copy (SSC), and two inverted repeat regions (IRa and IRb). The cp genome sizes ranged from 135,557 to 136,006 bp, with an average of 135,878 bp (Table 1). The 25 complete cp genome sequences and the generic (or consensus) “*Avena*” cp genome sequence are available on Supplementary File S1 and File S2, respectively. Changes in cp genome size mainly were reflected in the LSC region. The average sizes for LSC, SSC, and IR among the 25 *Avena* species were 80k bp, 12.6k bp, and 21.6 kb, respectively. The average of GC content ranged from 38.41% to 38.53%, with an average of 38.49% (Table 1).

### 2.2. Chloroplast Genome Gene Annotation

The gene annotations revealed an identical set of 130 cp genes for these 25 oat species (Table 2; Supplementary File S3). Of all the 130 genes, 84 coding genes, eight rRNA, and 38 tRNA were identified as common genes across the oat species of variable ploidy (Table 2; Figure 1). There were 21 genes (13 coding genes and eight tRNA genes) that had at least one intron. Also, four pseudogenes were found in the two diploid species (*A. clauda* and *A. eriantha*), and six pseudogenes were identified for the other 23 oat species. All pseudogenes were distributed in the LSC region. Both IRa and IRb regions shared the same amount of 19 duplicated genes, but one marked difference is that the *ndhH* gene had different C terminals. As both 3’ ends of the *ndhH* genes were extended to either side of the SSC fragment, it caused a different translation of peptide tails. Two genes *infA* and *rps16*, coding for a translation initiation factor 1 and a S16 ribosomal protein, respectively, were detected in these oat species.

### 2.3. Comparison of Genomic Structures

**Figure 2:**
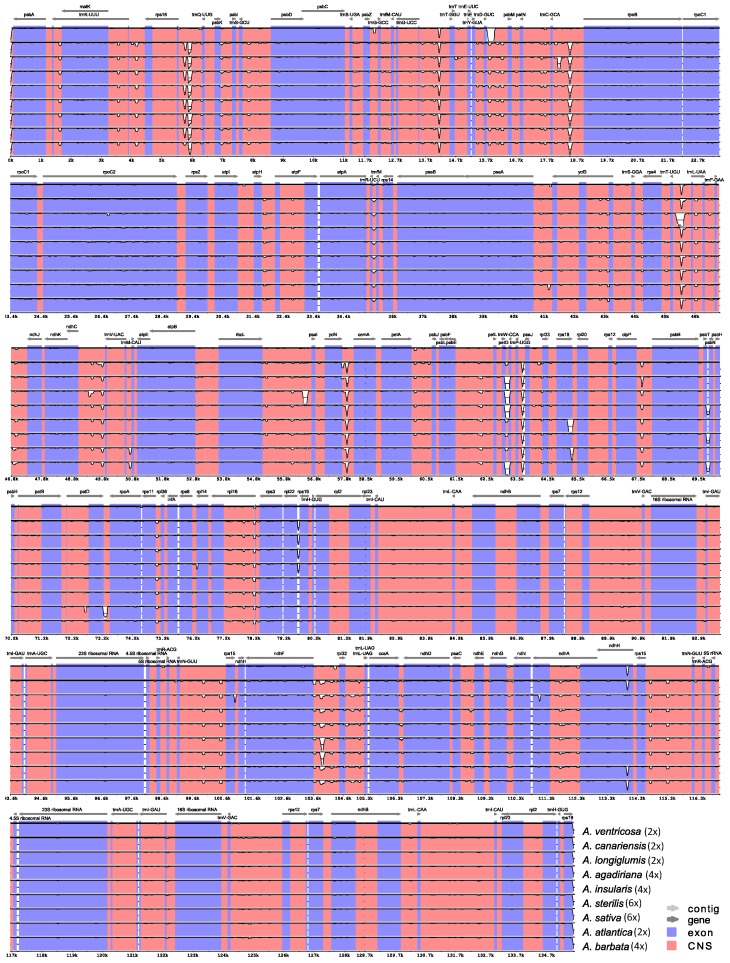
Percent identity plot by mVISTA of nine *Avena* chloroplast genome assemblies representing four diploid, three tetraploid, and two hexaploid *Avena* species, using *A. eriantha* as reference. Vertical scale indicates the percentage of identity ranging from 98.385% to 99.377%. Coding regions are in blue and non-coding regions are in orange.

Analyzing mVISTA percent identity plot revealed several major features of genomic variation, as illustrated in Figure 2 for nine oat species (and Supplementary File S4 for 25 oat species). First, no marked differences in genomic structure and gene arrangement were observed. Second, the degree of similarity between any two of 25 cp genome sequences ranged from 98.380% to 99.996%, with an average of 99.529%. Third, most of the nucleotide variations across the 25 cp genomes were located in intergenic regions. Fourth, sequence variation among the 25 genomes was also identified for the *ndhH* gene. Fifth, there were no specific variations in genomic structure and gene arrangement unique to each ploidy level.

### 2.4. Sequence Variation and Divergence

The SNP calls based on the full length of 25 cp genome sequence alignments with the most stringent conditions revealed a total of 1313 SNPs for these 25 species (see Supplementary File S5). There were 583 SNPs (44.4%) located in the genic regions, 16 SNPs (1.2%) in the pseudogene, and 714 SNPs (54.4%) distributed in the intergenic regions.

The simple sequence repeat (SSR) analysis revealed considerable SSR polymorphism in these 25 oat cp genomes. A total of 6694 SSRs were identified for the 25 oat species. The SSR counts per species ranged from 256 (*A. clauda* and *A. eriantha*) to 280 (*A. atlantica*), with an average of 276.8. The SSR motifs were mainly poly-A, with eight to 18 repeats; poly-C, with eight to 14 repeats; poly-AT, with five to seven repeats; and poly-AG with five repeats (Table 3). Six abundant SSR motifs were poly-A, with 8, 9, 10, and 11 repeats, followed by poly-C, with eight repeats and poly-AT, with five repeats. However, no SSR motifs for tri-, tetra, pentra-, and hexa-nucleotides were found.

**Figure 3:**
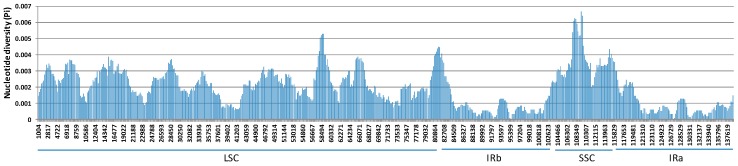
Nucleotide diversity (Pi) from the sliding window analysis of 25 complete *Avena* chloroplast genome assemblies (window length: 2000 bp, step size 200 bp). X-axis: position of the window midpoint, Y-axis: nucleotide diversity within each window.

The sliding window analysis of nucleotide diversity showed that the genomic region with the highest nucleotide diversity was SSC, followed by LSC, while two repeat regions (IRa and IRb) had the lowest nucleotide diversity (Figure 3). Three specific genome positions with the highest diversity were the sliding windows near 108,349, 59,494, and 81,000 (Figure 3).

### 2.5. Selective Pressure Analysis

The positive selection analysis considered all 130 genes, with a total of 19,941 amino acids. The likelihood ratio tests for three models (M2a vs. M1a; M8 vs. M7; and M8 vs. M8a) appeared to suggest the presence of positive selections with p-values < 0.05 on many cp genes (Table 4). Based on the Naïve Empirical Bayes test, there were 114 codons and six codons showing positive selections with the posterior probability of 50% and 95%, respectively. However, if the Bayesian Empirical Bayes tests were used, there were only six codons showing positive selections with the posterior probability of 84% only. The six positively selected sites on the amino acids of six genes were 817 Gly (*matK*), 2150 Cys (*rpoB*), 4457 Val (*rpoC2*), 10,203 Glu (*rbcL*), 11,332 Leu (*rpl33*), and 16,682 Phe (*ccsA*). Additional maximum likelihood analysis of natural selection on individual codons identified the codon #17177 (CTA at the genome position of 55,930) for the amino acid leucine with the p-value of 0.0096. These results, together, indicate that positive selection was not strong and purifying selection was dominant, acting on these 25 oat cp genomes.

## 3. Discussion

Our cp genomic analysis here generated the first set of cp genome assemblies for these 25 oat species. The genomes typically had four regions (LSC, SSC, IRa, and IRb), with lengths of roughly 136,006 bp, and they carried 130 genes and four to six pseudogenes. Purifying selection was the dominant force acting on the cp genes. The genomes harbored 1313 SNPs and 277 SSRs per species. More nucleotide diversity was located in the SSR and LSC, rather than IRa and IRb, regions. These research outputs allowed for a better understanding of oat cp genomes and evolution, and they formed an essential set of cp genomic resources for future oat studies and assessing oat genetic resources.

The analysis also revealed many comparative characteristics and some unique features of these oat cp genomes. First, the two genes *infA* and *rps16*, coding for a translation initiation factor 1 and a S16 ribosomal protein, respectively, were detected in these oat species, like other grass species, such as wheat and barley, but they were reported to be absent or nonfunctional in Malpighiales: *Passiflora edulis*, *Jatropha curcas*, and *Manihot esculenta* [29,30,31]. Second, a large sequence variation among 25 cp genomes was identified for the *ndhH* gene. Third, little differences in genomic structure and gene arrangement were identified across 25 species, and no marked genomic variations were unique to an oat ploidy level. Fourth, the positive selection was relatively weak acting on the 130 cp genes.

These comparative genomic features provided some empirical support for the previous inferences of oat evolution [9,18], as these genomes had the same gene count and arrangement, and purifying selection was the dominant force of selection acted on most of the 130 genes. Two C-genome species (*A. clauda* and *A. eriantha*) had only four pseudogenes, while the other 23 species had six. Such variation might be related to the major divergence of C-genome species 13–15 million years ago from A-genome species [18]. The indel variations for the *ndhH* gene seemed to be associated with the divergence of the As- and AB-genomes, but not with the divergence of hexaploid oat lineage. The cp genomic variations appeared to be not associated with the nuclear genome polyploidizations.

Managing more than 13,000 accessions of oat wild relatives is a challenging task, as many difficult issues must be properly addressed, such as taxonomic delimitation, duplication identification, viability monitoring, and field regeneration [17,32]. The cp genomic resources developed here can be utilized to develop taxonomic barcodes [8] for germplasm identification, e.g., from the region between the *psbZ* and *trnfM* (CAU) genes (see Supplementary File S4). Specific barcoding to identify wild relative germplasm of specific interests such as ecological types and geographic origin can also be developed. Molecular characterization of wild relative germplasm using cp markers, such as SNPs or SSRs, may be more informative than those using nuclear genomic tools, as cp markers are more conservative and nuclear genomic markers developed from polyploidy plants may not necessarily be informative. The inferred maternal phylogeny from these cp genomic resources (e.g., see [18]) can provide some guide for the search of evolutionarily related, economically important cp genes for gene introgression into oat breeding programs [15,16] through cp genome engineering [10]. The cp genome sequences are also essential for selecting intergenic spacer regions in oat cp genomes for transgene integration and assessing cp genome regulatory sequences in transgene expression [10]. We have initiated research to utilize these developed genomic resources to enhance the conservation, management and utilization of the oat collection in Plant Gene Resources of Canada.

## 4. Materials and Methods

### 4.1. Plant Material

We selected 25 *Avena* accessions of known species identity from the Plant Gene Resources of Canada (PGRC) oat collection, based on our previous oat research [3]. The selected accessions originated from various regions around the world and represent 25 species of the six botanical sections of the *Avena* genus, Ventricosa, Agraria, Tenuicarpa, Pachycarpa, Ethiopica, and Avena, and five distinct nuclear genomes organized in diploid (A or C), tetraploid (AB or AC), and hexaploid species (ACD). Table 1 shows the detailed information on the selected accessions with PGRC accession numbers, including botanical section and ploidy. About 300 seeds from each accession were planted in August 2013 in a 15 cm pot, grown for 8 to 10 days in the greenhouse at Saskatoon Research and Development Centre of Agriculture and Agri-Food Canada, and then incubated in the dark for 48 to 72 h. Up to 15 g from all 300 seedling leaves were collected and washed in cold water. Leaves were cut into 1 cm pieces with scissors, snap frozen with liquid nitrogen in a –20°C mortar, and then ground to a fine powder. Ground samples, while still frozen, were transferred to 50 mL conical-bottom centrifuge tubes, cooled on dry ice, and then stored at –80°C, for up to one week.

### 4.2. DNA Extraction and MiSeq Sequencing

Plastid DNA isolation was performed following the method of Shi et al. [33] and optimized using the cp DNA extraction protocol developed by Diekmann et al. [34]. All the procedures were carried out on ice or at 4°C with buffers prechilled to 4°C. The enriched cp pellet was allowed to thaw at room temperature, and DNA was extracted using the Qiagen DNEasy Plant Mini kit standard method on a Qiacube robot (Qiagen, Mississauga, Canada) and eluted in 1/3x Qiagen AE buffer (3.33 mM Tris-Cl, 0.17 mM EDTA, pH9.0). DNA samples were quantified using the Quant-iT PicoGreen dsDNA Assay Kit (Life Technologies, Burlington, Canada). Final DNA yields ranged from 0.2 to 4.3 ng/µl and were diluted to 0.2 ng/µl with 10 mM Tris-HCl, with a pH of 8. The acquired cp DNAs were subjected to the genomic DNA library preparation with a Nextera XT DNA Library Preparation Kit (Illumina). Four MiSeq runs, each with six or seven libraries and pair-end of 250 bp, were performed to generate 25 forward and 25 reverse FASTQ files. All raw reads were deposited into the National Center for Biotechnology Information (NCBI), under the Bioproject PRJNA401438 (Table 1).

### 4.3. Chloroplast Genome Assembling and Annotation

All raw sequence reads were cleaned first with cutadapt [35] to remove sequence adapters and to perform quality trimming. Partial Nextera adapter sequence ‘AGATGTGTATAAGAGACAG’ was used to trim the raw sequence reads. All the sequence reads with both quality lower than 15 and shorter than 150 bp were discarded. SPAdes v3.11.1 [36] was used as the assembler for the circular cp genome assembly in the pair-end mode. Preliminary tests were performed to reach the least contigs number and the longest scaffold size by a series of combinations of different coverages and k-mer sizes. The k-mer size was eventually set to 127, and the coverage was set to 1000 folds, after a series of training analyses. The four major gaps located at the four junctions (LSC-IR, IR-SSC, SSC-IR, and IR-LSC) were filled in by the assistance of the four junction sequences. These junction sequences were obtained from the alignments of the scaffolds with their closely related species, including wheat (*Triticum aestivum*, NCBI Reference Sequence: AB042240.3; [37]), bent grass (*Agrostis stolonifera*; NCBI Reference Sequence: NC_008591.1), and ryegrass (*Lolium perenne*; NCBI Reference Sequence: NC_009950.1) cp genome. Each of the four junction sequences (ranging between 540 and 700 bp) containing both IR and another (either LSC or SSC) structure fragment was used as a bait to screen for reads for further gap sequence recovery. The selected reads from BLAST were also used to link adjacent structure fragments. The additional gaps located within the scaffolds of several *Avena* accessions were similarly filled with the assistance of the bait sequences acquired from either wheat or other *Avena* cp genomes with sequences at the same locations.

Gene annotations of 25 cp genomes were made using online DOGMA program [38], along with the cp genome annotations of wheat (NCBI Reference Sequence: AB042240), ryegrass (NCBI Reference Sequence: NC_009950), and bent grass (NCBI Reference Sequence: NC_008591.1). Manual curation was also made for the variations within coding genes, such as rRNA and tRNA, based on multiple sequence alignments with their closely related species in the Triticeae tribe. The circular maps of the *Avena* cp genomes were generated first with GenomeVx [39] and Circos [40] and finally merged in Inkscape (https://inkscape.org).

### 4.4. Comparative Genomic Analysis

To identify the genomic regions with substantial variability, the complete cp genomes of 25 *Avena* species were compared using mVISTA [41], with wheat cp genome as reference. For this comparison, the percent identity matrix among 25 cp genomes was also generated. To illustrate the genomic variations with respect to ploidy, further effort was also made, using *A. eriantha* cp genome as reference to compare nine cp genomes, representing diploid, tetraploid, and hexaploid species.

### 4.5. SNP, SSR, and Diversity Analysis

The SNP calling was performed on the basis of multiple sequence alignments (MSA) by SNP-sites with the default options [42]. MAFFT was used to generate MSA data with the FFT-NS-i×1000 alignment algorithm [43]. To identify SSRs, 25 cp genomes were analyzed using MISA [44], with the following setting of minimum numbers of repeats to 8, 5, 4, 3, 3, and 3 for mono-, di-, tri-, tetra, pentra-, and hexa-nucleotides, respectively. The sliding window diversity analysis was done using DnaSP v6 [45] to estimate nucleotide diversity across 25 oat species, with a sliding window of 2000 bp and step size of 200 bp.

### 4.6. Selective Pressure Analysis

We applied several site models (M0, M1, M2, M3, M7, and M8), implemented in codeml of PAML v 4.9i [46], to estimate the Ka/Ks and ω values, considering F3X4 codon frequencies. MAFFT, with the default options, was used to align the nucleotide sequences of all cp genes, and the phylogenetic tree of 25 *Avena* species, without tree-branch lengths, was obtained from the previous phylogenetic analysis [18]. Four nested site models (M3 vs. M0; M2 vs. M1; M8 vs. M7; and M8a vs. M8) were evaluated by log-likelihood ratio tests (LRT). The positively selected sites were analyzed by Naïve Empirical Bayes (NEB) analysis and Bayesian Empirical Bayes (BEB) analysis. Extra effort was also made to perform a maximum likelihood analysis of natural selection codon-by-codon, using MEGA 7 [47], following the method of Suzuki and Gojobori [48].

## Figures and Tables

**Table 1:** List of 25 studied *Avena* species from six botanical sections and their cp genome assemblies.

Section/Species	Pl	Raw Reads	CPG Size (bp)	CPG Region (bp)				GC%	NCBI Acc#	PGRC Acc#
				LSC	IRb	SSC	IRa			
Ventricosa										
*A. ventricosa*	2x	3,399,167	135,681	79,793	21,614	12,660	21,614	38.41	MG687301	CN21992
*A. clauda*	2x	3,037,449	135,557	79,667	21,619	12,652	21,619	38.43	MG687303	CN19205
*A. eriantha*	2x	3,932,778	135,560	79,669	21,619	12,653	21,619	38.43	MG687291	CN19256
Agraria										
*A. hispanica*	2x	3,138,274	135,935	80,099	21,605	12,626	21,605	38.49	MG687300	CN25788
*A. brevis*	2x	3,145,903	135,939	80,101	21,606	12,626	21,606	38.49	MG687310	CN3145
*A. nuda*	2x	3,342,392	135,934	80,100	21,604	12,626	21,604	38.48	MG687306	CN79350
*A. strigosa*	2x	2,618,401	135,938	80,102	21,605	12,626	21,605	38.48	MG687309	CN22002
Tenuicarpa										
*A. canariensis*	2x	4,285,394	135,955	80,147	21,598	12,612	21,598	38.52	MG687297	CN25449
*A. damascena*	2x	2,906,067	135,925	80,101	21,602	12,620	21,602	38.51	MG687302	CN19458
*A. atlantica*	2x	4,092,127	136,006	80,168	21,606	12,626	21,606	38.48	MG687299	CN25859
*A. wiestii*	2x	4,372,976	135,944	80,109	21,605	12,625	21,605	38.49	MG687296	CN24315
*A. lusitanica*	2x	4,425,054	135,879	80,166	21,603	12,507	21,603	38.52	MG687295	CN25936
*A. longiglumis*	2x	4,288,420	135,728	79,881	21,605	12,637	21,605	38.53	MG687305	CN21407
*A. agadiriana*	4x	4,432,963	135,945	80,129	21,602	12,612	21,602	38.49	MG687294	CN25868
*A. barbata*	4x	4,362,663	135,946	80,111	21,605	12,625	21,605	38.49	MG687311	CN24462
Pachycarpa										
*A. insularis*	4x	4,001,844	135,967	80,130	21,603	12,631	21,603	38.5	MG674209	CN19178
*A. maroccana*	4x	4,160,684	135,887	80,102	21,604	12,577	21,604	38.51	MG687298	CN23057
*A. murphyi*	4x	5,199,242	135,892	80,108	21,604	12,576	21,604	38.51	MG687312	CN21989
Ethiopica										
*A. vaviloviana*	4x	3,870,454	135,946	80,111	21,605	12,625	21,605	38.49	MG687304	CN22413
*A. abyssinica*	4x	2,822,158	135,942	80,109	21,604	12,625	21,604	38.49	MG687293	CN22064
Avena										
*A. fatua*	6x	4,120,412	135,889	80,106	21,604	12,575	21,604	38.51	MG687307	CN21948
*A. hybrida*	6x	4,330,653	135,900	80,117	21,604	12,575	21,604	38.51	MG687292	CN24926
*A. occidentalis*	6x	2,947,248	135,893	80,114	21,602	12,575	21,602	38.51	MG687314	CN25946
*A. sterilis*	6x	2,467,386	135,888	80,107	21,603	12,575	21,603	38.51	MG687308	CN20625
*A. sativa*	6x	5,329,202	135,886	80,107	21,602	12,575	21,602	38.51	MG687313	CN24549

Note: Pl is for ploidy level. CPG is chloroplast genome. LSC, SSC, and IR are large single copy, small single copy and inverted repeat regions, respectively. NCBI Acc# are the accession numbers for the cp assemblies deposited in the National Center for Biotechnology Information (NCBI). PGRC Acc# are the accession numbers for the studied samples obtained from the oat collection at Plant Gene Resources of Canada (PGRC).

**Table 2:** List of 130 genes and their duplications found in the plastids of 25 *Avena* species.

Category	Gene*
Subunits of photosystem I	*psaA, psaB, psaC, psaI, psaJ*
Subunits of photosystem II	*psbA, psbB, psbC, psbD, psbE, psbF, psbH, psbI, psbJ, psbK, psbL, psbM, psbN, psbT, psbZ*
Subunits of cytochrome b/f complex	*petA, petB^a^, petD^a^, petG, petL, petN*
Subunits of ATP synthase	*atpA, atpB, atpE, atpF^a^, atpH, atpI*
Large subunit of rubisco	*rbcL*
Subunits of NADH-dehydrogenase	*ndhA^a^, ndhB(1)^a^, ndhC, ndhD, ndhE, ndhF, ndhG, ndhH(1), ndhI, ndhJ, ndhK*
Proteins of large ribosomal subunit	*rpl2(1)^a^, rpl14, rpl16^a^, rpl20, rpl22, rpl23(1), rpl32, rpl33, rpl36*
Proteins of small ribosomal subunit	*rps2, rps3, rps4, rps7(1), rps8, rps11, rps12(1)^a^, rps14, rps15(1), rps16^a^, rps18, rps19(1)*
Subunits of RNA polymerase	*rpoA, rpoB, rpoC1, rpoC2*
Cytochrome c biogenesis	*ccsA*
Transfer RNAs	*tRNA-Ala(2), tRNA-Arg(3), tRNA-Asn(2), tRNA-Asp, tRNA-Cys, tRNA-Gln, tRNA-Glu, tRNA-Gly(2), tRNA-His(2), tRNA-Ile(4), tRNA-Leu(4), tRNA-Lys, tRNA-Met(2), tRNA-Phe, tRNA-Pro, tRNA-Ser(3), tRNA-Thr(2), tRNA-Trp, tRNA-Tyr, tRNA-Val(3)*
Ribosomal RNAs	*16S rRNA(2), 23S rRNA(2), 4.5S rRNA(2), 5S rRNA(2)*
Maturase	*matK*
Protease	*clpP*
Conserved hypothetical genes	*ycf3^a^, ycf4*
Envelope membrane protein	*cemA*
Translation initiation factor	*infA*

* The superscript ^a^ means the gene contains intron(s). The number in parentheses after a gene shows the number of duplications for the gene in the other genome regions.

**Table 3:** Simple sequence repeat (SSR) polymorphism found in the plastids of 25 *Avena* species.

SSR type	A	A	A	A	A	A	A	A	A	A	A	C	C	C	C	C	C	AT	AT	AT	AG
Repeat count	8	9	10	11	12	13	14	15	16	17	18	8	9	10	11	12	14	5	6	7	5
*A. ventricosa*	62	33	14	5	1	1	2	0	0	0	1	4	3	1	0	0	0	4	0	1	1
*A. clauda*	58	32	12	8	1	1	2	0	0	0	1	3	1	3	0	1	0	3	1	0	1
*A. eriantha*	57	30	15	8	1	1	2	0	0	0	1	3	1	3	1	0	0	3	1	0	1
*A. hispanica*	67	27	17	6	1	1	1	1	1	0	0	7	4	0	0	0	0	4	1	0	1
*A. brevis*	67	27	16	7	1	0	2	1	1	0	0	7	2	2	0	0	0	4	1	0	1
*A. nuda*	67	29	15	5	1	1	2	1	1	0	0	9	2	0	0	0	0	4	1	0	1
*A. strigosa*	67	29	15	2	4	2	1	1	1	0	0	7	4	0	0	0	0	4	1	0	1
*A. canariensis*	66	29	17	4	1	0	1	0	1	0	0	6	2	2	0	0	0	3	0	1	1
*A. damascena*	66	27	13	7	4	1	1	0	0	1	0	5	4	0	0	0	1	3	0	1	1
*A. atlantica*	68	30	15	2	2	2	3	1	0	0	0	7	2	2	0	0	0	4	1	0	1
*A. wiestii*	66	28	14	7	1	0	1	1	2	0	0	7	1	2	1	0	0	4	1	0	1
*A. lusitanica*	63	26	17	8	1	1	1	0	1	0	0	6	1	3	0	0	0	3	0	1	1
*A. longiglumis*	63	28	12	9	3	0	2	0	0	0	0	7	1	0	2	0	0	3	0	1	1
*A. agadiriana*	62	29	12	9	2	0	1	1	0	0	0	5	4	0	0	0	0	3	0	1	1
*A. barbata*	66	27	14	7	2	0	1	2	1	0	0	7	1	2	1	0	0	4	1	0	1
*A. insularis*	63	30	11	8	2	0	2	0	1	0	0	6	1	3	0	0	0	3	0	1	1
*A. maroccana*	66	29	11	8	1	0	2	0	0	0	0	5	3	0	2	0	0	3	1	0	1
*A. murphyi*	63	29	12	8	2	0	1	1	0	0	0	7	0	1	2	0	0	3	1	0	1
*A. vaviloviana*	66	27	13	9	1	0	1	1	2	0	0	7	1	2	1	0	0	4	1	0	1
*A. abyssinica*	66	27	13	10	0	0	2	1	1	0	0	7	3	0	1	0	0	4	1	0	1
*A. fatua*	64	30	9	9	2	0	1	1	0	0	0	7	1	0	2	0	0	3	1	0	1
*A. hybrida*	64	30	11	8	1	0	2	0	0	0	0	7	0	1	2	0	0	3	1	0	1
*A. occidentalis*	64	32	10	7	1	0	2	0	0	0	0	7	2	1	0	0	0	3	1	0	1
*A. sterilis*	64	29	9	11	1	0	1	1	0	0	0	7	1	2	0	0	0	3	1	0	1
*A. sativa*	64	29	10	9	2	0	1	1	0	0	0	7	3	0	0	0	0	3	1	0	1
Mean	64.4	28.9	13.1	7.2	1.6	0.4	1.5	0.6	0.5	0.0	0.1	6.3	1.9	1.2	0.6	0.0	0.0	3.4	0.7	0.3	1.0

**Table 4:** Log-likelihood values (InL) and parameter estimates under models of variable ω ratios among sites of 19,941 codons in 25 *Avena* chloroplast genomes.

Model Code	InL	p-value	Estimates of Parameters	Count of PSS^b^	
		for LRT^a^		NEB	BEB
M0 (One-Ratio)	–82795.020		ω = 0.11878		
M3 (Discrete)	–82781.913	0.000013	p0 = 0.10199, p1 = 0.85429, (p2 = 0.04372),	0(114)	
			ω0 = 0.00000, ω1 = 0.00000, ω3 = 2.81808		
M1a (Nearly neutral)	–82785.669		p0 = 0.90153, (p1 = 0.09847), ω0 = 0.00000, (ω1 = 1.00000)		
M2a (Positive selection)	–82781.919	0.011758	p0 = 0.94578, p1 = 0.02070, (p2 = 0.03352),	114(0)	6(0)
			ω0 = 0.00110, (ω1 = 1.00000), ω2 = 2.98551		
M7 (Beta)	–82784.258		p = 0.00500, q = 0.06086		
M8 (Beta and ω)	–82781.940	0.049250	p0 = 0.97686, p = 0.05383, q = 1.11417, (p1=0.02314), ω=3.81079	114(6)	6(0)
M8a (Beta and ω > 1)	–82787.155		p0 = 0.95611, p = 0.07461, q = 0.93085, (p1 = 0.04389), ω = 1.00000		
M8 (Beta and ω)	–82781.940	0.000671	p0 = 0.97686, p = 0.05383, q = 1.11417, (p1 = 0.02314), ω = 3.81079	114(6)	6(0)

^a^ LRT = likelihood ration test. ^b^ PSS = positively selected site; NEB = Naïve Empirical Byes analysis; BEB = Bayesian Empirical Bayes analysis; and the first number is the count of PSS with posterior probabilities >50%, and the second number (in parenthesis) is the count of PSS with posterior probabilities >95%.

## Supplementary Materials

The supplementary materials consist of five files as below. These files are accessible online on FigShare (DOI://10.6084/m9.figshare.9759449). File S1: A FASTA file for 25 complete *Avena* cp genome sequences; File S2: A FASTA file for a consensus genome sequence from 25 *Avena* cp genomes; File S3: An Excel file for gene annotation feature table; File S4: A pdf file for percent identity plot of 25 *Avena* cp genomes; File S5: An Excel file for 1313 cp SNPs across 25 cp genomes.
